# Impact of Maternal Weight Gain on the Newborn Metabolome

**DOI:** 10.3390/metabo13040561

**Published:** 2023-04-15

**Authors:** Teresa Guixeres-Esteve, Francisco Ponce-Zanón, José Manuel Morales, Empar Lurbe, Julio Alvarez-Pitti, Daniel Monleón

**Affiliations:** 1 Pediatric Department, Consorcio Hospital General, University of Valencia, 46014 Valencia, Spain; maguies3@uv.es (T.G.-E.);; 2 INCLIVA Biomedical Research Institute, Hospital Clínico, University of Valencia, 46010 Valencia, Spain; 3 Biomedical Research Networking Center for Physiopathology of Obesity and Nutrition (CIBEROBN), Institute of Health Carlos III (ISCIII), 28029 Madrid, Spain; 4 Department of Pathology, University of Valencia, 46010 Valencia, Spain; 5 CIBER of Frailty and Healthy Aging (CIBERFES), Institute of Health Carlos III (ISCIII), 28029 Madrid, Spain

**Keywords:** metabolomics, gestational weight gain, offspring, newborn, umbilical cord

## Abstract

Pre-pregnancy obesity and excessive gestational weight gain (GWG) appear to affect birth weight and the offspring’s risk of obesity and disease later in life. However, the identification of the mediators of this relationship, could be of clinical interest, taking into account the presence of other confounding factors, such as genetics and other shared influences. The aim of this study was to evaluate the metabolomic profiles of infants at birth (cord blood) and 6 and 12 months after birth to identify offspring metabolites associated with maternal GWG. Nuclear Magnetic Resonance (NMR) metabolic profiles were measured in 154 plasma samples from newborns (82 cord blood samples) and in 46 and 26 of these samples at 6 months and 12 months of age, respectively. The levels of relative abundance of 73 metabolomic parameters were determined in all the samples. We performed univariate and machine-learning analysis of the association between the metabolic levels and maternal weight gain adjusted for mother‘s age, Body Mass Index (BMI), diabetes, diet adherence and infant sex. Overall, our results showed differences, both at the univariate level and in the machine-learning models, between the offspring, according to the tertiles of maternal weight gain. Some of these differences were resolved at 6 and 12 months of age, whereas some others remained. Lactate and leucine were the metabolites with the strongest and longest association with maternal weight gain during pregnancy. Leucine, as well as other significant metabolites, have been associated in the past with metabolic wellness in both general and obese populations. Our results suggest that the metabolic changes associated to excessive GWG are present in children from early life.

## 1. Introduction

In 1989, Barker et al. hypothesized that an unfavorable environment in utero can cause programmed adaptations in fetal development that persist into extrauterine life, creating a phenotype that is more susceptible to cardiovascular disease [1]. An example of an unfavorable intrauterine milieu is fetal overnutrition, defined as fetal exposure to excess maternal fuels due to maternal obesity, gestational diabetes (GD), or excessive gestational weight gain (GWG). Many epidemiological, clinical, and animal-model studies strongly suggest that mothers’ prenatal obesity and high-fat dietary intake are associated with cardiometabolic morbidity in their progeny, including obesity, hypertension, hyperglycemia and insulin resistance, hyperlipidemia and non-alcoholic fatty-liver disease [2,3].

Many studies suggest that cord-plasma-metabolite profiles (resulting from fetal response to in utero exposure) can serve as early-life biomarkers of the risk of metabolic disease in the long term. Hence, interest has grown in the investigation of metabolomic profiles in the cord blood of mother-child pairs with inconsistent results produced by the studies conducted thus far.

Perng et al. reported that the metabolites in energy-production and DNA/RNA turnover pathways and the branched-chain amino acids (BCAAs) in cord blood were associated with larger neonatal size (a known risk factor for poor cardiovascular health later in life), and that BCAAs and androgen hormone patterns were higher in obese compared with lean school-age children (6–10 years). Furthermore, higher factor scores for these patterns were correlated with continuous measures of overall and central adiposity as wellas other cardiometabolic biomarkers, such as higher HOMA-IR [4,5]. Kadakia et al. found that cord-blood BCAAs and ketone-body metabolites were positively correlated with newborn adiposity [6]. Cao et al. linked cord-plasma metabolites with the longitudinal BMI trajectories of 946 children from birth up to the age of 18 years. These trajectories were categorized into three patterns: early-onset overweight and obesity (early-OWO), late-onset OWO (late-OWO), and normal weight trajectory (NW). These results suggested that the cord-blood metabolome was most useful factor in identifying children at risk of early-OWO, with twenty-two triacylglycerols and diacylglycerols negatively associated with early-OWO, and five cholesterol esters positively associated [7].

Similarly, Perng et al. did not find any significant associations between cord-blood-metabolite patterns and maternal characteristics, except for cesarean delivery, some metabolites of energy production, and cell-proliferation pathways, which were associated with larger size at birth. On the other hand, they observed that the BCAA scores were higher in the cord plasma of overweight women before pregnancy [5]. Lowe et al. found an association between maternal BMI and cord-plasma levels of BCAAs, their metabolic bioproducts, and phenylalanine [8]. Francis et al. noted differences in fifty-two metabolites between infants who were exposed to fetal overnutrition and those who were not exposed, with a small amount of variation between maternal obesity only, GD only, and both [9].

However, while obesity and GD are widely studied as conditions in fetal overnutrition, few metabolomic studies focus on GWG. To gain a better understanding of the latter’s influence, we performed a longitudinal study on obese and lean pregnant women classified by tertiles of GWG. The aim of our study was to identify differences between offspring metabolite profiles, from the newborn (cord blood) to the 12-months stage, with respect to maternal GWG.

## 2. Materials and Methods

### 2.1. Study Population: Clinical and Biochemical Measurements

This study hypothesized that maternal-dependent factors, especially GWG, lead to metabolomic changes in newborns and during the first year of life.

For this purpose, a prospective observational cohort study was designed, beginning at birth and following up to 12 months of life. Eighty-three mother-newborn pairs were divided into three groups, according to tertiles of weight gain, to analyze the impact of the mother’s gestational weight gain. To participate in the study, the following inclusion and exclusion criteria were set:: newborns born at full term (over 37 in weeks gestational age at birth), ascertained according to the Ballard method [10], by normal delivery or by cesarean section, at the Hospital General Universitario of Valencia, Spain. Preterm newborns or newborns with any perinatal pathology were excluded. Mothers included were normal weight, obese or obese with gestational diabetes except mothers with gestational diabetes treated with insulin or any other illness.

All participating mothers received written information about the study and signed their informed consent to participate. The study was approved by the hospital’s review board and was carried out in accordance with the Declaration of Helsinki. Both the samples (plasma from umbilical cord of the newborns and plasma from peripheral veins from the mothers), and the collected data were stored according to the directives dictated by the law of Biomedical Investigation of 2007 (Law 14/2007) and all applicable rules.

(A) From mothers, age, height, and weight measurements prior to gestation as recorded in their pregnancy booklet by the midwife, and weight at the end of gestation were collected. The GWG was calculated and its conformity with the 2009 recommendations of the Institute of Medicine (IOM) was verified [11]. Regarding the evolution of the pregnancy, the presence or absence of gestational diabetes, the degree of maternal adherence to the Mediterranean diet (AMD) using the validated questionnaire developed by Trichopoulos et al. [12] and smoking habits were recorded. Finally, the type of delivery was also recorded (vaginal or cesarean section).
(B) From all newborns, the following information was recorded:
  - Somatometry (weight, length, head circumference and ponderal index and percentiles according to Intergrowth-21st), performed by trained nurses in the maternity ward. Weight was measured with ADE scale model M112600 (ADE GmbH & Co, Hamburg, Germany). Length was measured in the supine position using a neonatometer. Head circumference was measured with a tape measure at the maximum circumference. The ponderal index, also known as the corpulence index or Rohrer’s index, was calculated with the following formula PI = weight/length^3^ × 100. Newborns were classified as small for gestational age (SGA), appropriate for gestational age (AGA) or large for gestational age (LGA) [13].
  - Blood pressure (BP) was obtained by taking 3 measurements, using oscillometric method, with System 7100 Non-invasive Blood Pressure AMI (Advanced Medical Instruments Inc., Broken Arrow, OK, USA) and heart rate (HR).
  - Type of feeding (breastfeeding or infant formula feeding); as well as the need for supplementation with artificial formula during the first days of life in those who were breastfed.
  - Umbilical cord blood samples were obtained from the clamped umbilical cord immediately after delivery for metabolomic studies.
(C) Blood sample were collected at 6 months of age to conduct metabolomic study.
(D) In infants, at 12 months of life, weight, length, head circumference and BMI were recorded, and their percentiles were calculated according to the WHO 2006/2007 curves [14]. The type of feeding at 12 months was recorded. Finally, blood samples were collected to perform metabolite and biochemical studies.

### 2.2. NMR Metabolomics

Umbilical-venous-cord blood was collected in EDTA tubes, centrifuged to yield plasma, stored at −80 °C and thawed before use. For NMR analysis, 500 μl of plasma was mixed with 50 μl of D2O (as a field lock). A total of 500 μl of the mixture of each sample was then individually transferred into a 5-mm high-quality NMR tube. A single-pulse presaturation NMR spectrum was acquired from all samples, with 256 transients collected into 65 k data points for all experiments. Nuclear magnetic resonance is a reproducible and accessible technique that has been applied successfully to the metabolic profiling of a variety of samples [15,16,17]. Spectra were processed using MestReNova 8.1 (Mestrelab Research S.L., A Coruña, Spain). They were then chemical-shift-referenced on the alanine CH3 doublet signal at 1.475 ppm, normalized to the total aliphatic area, lipid excluded, and transferred to MATLAB (MathWorks, Natick, MA, USA, 2012). We analyzed the chemical-shift region, including resonances of 0.50–4.70 ppm (the aliphatic region) and 5.20–10.00 ppm (the aromatic region). Resonances were annotated by research data and Chenomx resonances database (Chenomx NMR 7.6). The NMR peaks were quantified using semi-automated in-house MATLAB peak-fitting routines. The final metabolite relative spectral abundance was calculated. Machine learning and regression models were calculated in R 4.1 with the package ‘mdatools’. Finally, each metabolic feature was normalized to the standard deviation in all the samples from the analysis group (birth, 6 months, or 12 months) to obtain z-scores.

Partial least-squares discriminant analysis (PLS-DA) was applied to maximize the separation between samples and to identify discriminant patterns [18]. The PLSDA models were designed for child sex and weight at birth and for maternal obesity, adherence to the Mediterranean diet, and diabetes by calculating a linear-regression model with these variables for each metabolic feature and using the residues for the PLS-DA analysis. A permutation test was performed to check the potential overfitting of the PLS-DA models. The chemometric models were cross-validated with 10-fold Venetian blind cross-validation. In each replicate, 10% of the data were left out of the training calculations and used as a test dataset. Variable importance in projections (VIP) coefficients were calculated for all the models to select spectral features with strongest contributions to the models. Spectral features with high VIP coefficients contributed more to class separation during analysis, while those with very small VIP coefficients provided little contribution to classification. A Metabolite Set Enrichment Analysis (MSEA) of metabolites with VIPs scores higher than 1 and *p*-values below 0.05 was performed with MetaboAnalyst and the Small Molecule Pathway Database (SMPDB). Metabolite set enrichment analysis is conceptually similar to Gene Set Enrichment Analysis. A collection of predefined metabolite sets is used by MSEA algorithms to rank the lists of metabolites obtained in the analysis. This prior knowledge about metabolite makes it possible to identify significant and coordinated changes in metabolic networks and obtain biological insights.

### 2.3. Statistical Analysis

#### Demographics and Clinical Data Comparison

The IBM SPSS Statistics v.26 statistical program was used for data analysis. First, the database was segmented according to the 3 study groups, and the Kolmogorov Smirnov normality test was performed to verify that the population sample conformed to a normal distribution for the quantitative variables in the 3 groups. When a normal distribution of variables was not observed in any of the groups, the means of the variables in the 3 groups were compared with the nonparametric Kruskal Wallis test. In cases in which a variable had a normal distribution in the 3 groups, the means of the variable in the 3 groups were compared with ANOVA. The data on the quantitative variables were summarized in simple statistics (mean ± standard deviation). Differences between qualitative variables were analyzed using the Chi-square test and the data were expressed as percentages.

## 3. Results

The samples were divided according to the tertiles of the mothers’ absolute GWG. The maternal characteristics were comparable among the three groups except for BMI prior to gestation which was higher in the group in the first tertile of GWG (*p* = 0.004) and GWG adequacy according to the Institute of Medicine’s recommendations [11], which was higher in the first group, as expected. Table 1 shows the clinical differences between the mothers.

There were no clinical differences between the offspring at birth according to the tertiles of the maternal GWG, as shown in Table 2.

Table 3 shows the characteristics of the children at 12 months of age according to the tertiles of the absolute maternal GWG. There were no clinical differences at 12 months of age except for length (*p* = 0.039). There were also no statistically significant differences between the children’s blood tests at 12 months of age.

We quantified 43 metabolic spectral features in 82 blood-serum samples from the newborns (0 months), 46 samples at 6 months of age, and 26 samples at 12 months. We analyzed the data by age, exploring the associations between the metabolic profiles and maternal weights at the three different timepoints, adjusting for sex, weight at birth, and lactation of the newborn, as well as for maternal obesity, maternal diabetes, and adherence to the Mediterranean diet. The adjusted results were calculated on the subset, with all the covariates measured, which included 56 samples at 0 months, 42 at 6 months and 26 at 12 months.

**Figure 1:**
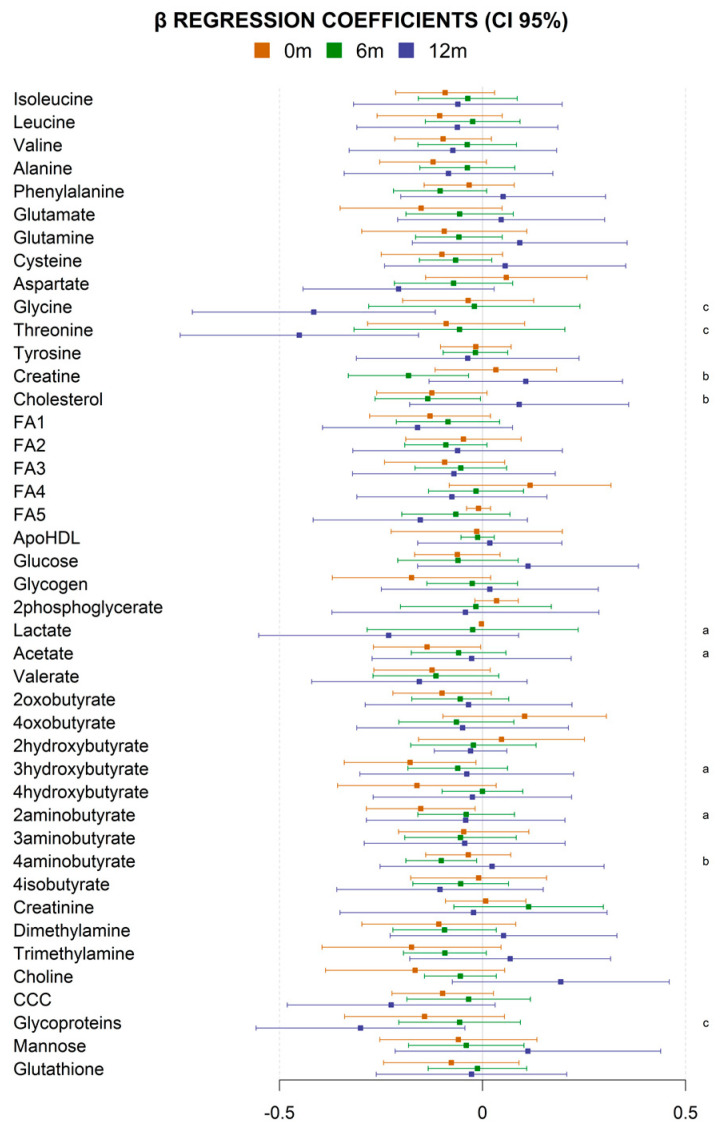
Linear association between maternal weight gain and metabolite levels. Linear regression beta coefficients and 95% confidence intervals (CI) for the association at birth (orange), 6 months of age (green), and 12 months of age (blue) between maternal weight gain and individual child serum metabolites adjusted for child sex, child weight at birth, maternal obesity, maternal diabetes, and maternal adherence to the Mediterranean diet. Each square represents the beta coefficient for a single metabolite, and the horizontal line indicates the 95% CI. Positive beta coefficients indicate a positive association with the outcome, while negative beta coefficients indicate a negative association. Metabolites with a statistically significant association (adjusted *p* value < 0.05) are marked as “a” (birth), “b” (6 months of age), and/or “c” (12 months of age). Label keys: CCC, choline-containing compounds; FA1, CH3 groups in fatty acids; FA2, beta CH2 groups in all, saturated and unsaturated, fatty acids; FA3, =CH_2_-CH_2_-CH_2_-CH= in fatty acids; FA4, =CH-CH_2_-CH= in fatty acids; FA5, all -CH=CH- in fatty acids; ApoHDL, apolipoproteins in HDL lipoparticles.

The adjusted linear regression between the metabolite levels and the maternal GWG was significant for different metabolites at different ages (Figure 1). Interestingly, the significant associations at birth were strongly dominated by short-chain fatty acids; this influence decreased at 6 months of age and disappeared at 12 months of age.

**Figure 2:**
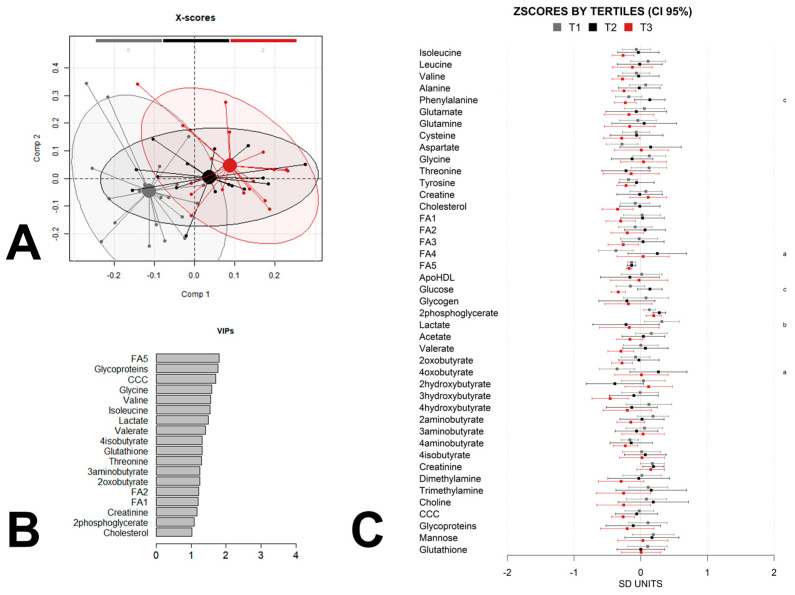
PLSDA analysis and z-scores of newborn metabolome at birth by maternal-weight gain tertile. (**A**) Score plot with 95% confidence ellipse of the PLS-DA analysis of the metabolome, adjusted for child sex, child weight at birth, maternal obesity, maternal diabetes, and maternal adherence to the Mediterranean diet for the discrimination between tertiles (tertile 1(T1) gray, tertile 2 (T2) black, tertile 3 (T3) red). Cross-validation parameters: RMSECV 0.304, R2CV: 0.582; ROC curve AUC: 0.96. (**B**) Metabolites with PLS-DA VIP score higher than 1 for the same PLS-DA metabolome model. (**C**) Z-scores and 95% confidence intervals of individual metabolites in offspring grouped by tertile. Metabolites with statistically significant pairwise differences between tertiles (adjusted *p*-value < 0.05) are labelled as “a” (tertile 1 vs. tertile2), “b” (tertile 1 vs. tertile 3), and/or “c” (tertile 2 vs. tertile 3). Label keys are the same as those in Figure 1.

**Figure 3:**
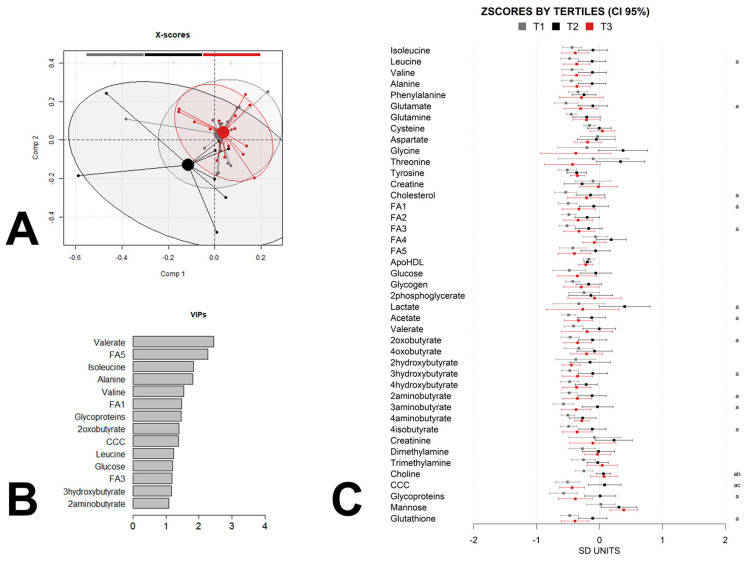
PLSDA analysis and z-scores of newborn metabolome at 6 months of age, by maternal-weight-gain tertile. (**A**) Score plot with 95% confidence ellipse of the PLS-DA analysis of the metabolome, adjusted for child sex, child weight at birth, maternal obesity, maternal diabetes, and maternal adherence to the Mediterranean diet for the discrimination between tertiles (tertile 1 (T1) gray, tertile 2 (T2) black, tertile 3 (T3) red). Cross-validation parameters: RMSECV 0.304, R2CV: 0.582; ROC Curve AUC: 0.96. (**B**) Metabolites with PLS-DA VIP score higher than 1 for the same PLS-DA model of the metabolome. (**C**) The Z-scores and 95% confidence intervals of individual metabolites in offspring grouped by tertile. Metabolites with statistically significant pairwise differences between tertiles (adjusted *p*-value < 0.05) are labeled as “a” (tertile 1 vs. tertile 2), “b” (tertile 1 vs. tertile 3), and/or “c” (tertile 2 vs. tertile 3). Label keys are the same as those in Figure 1.

**Figure 4:**
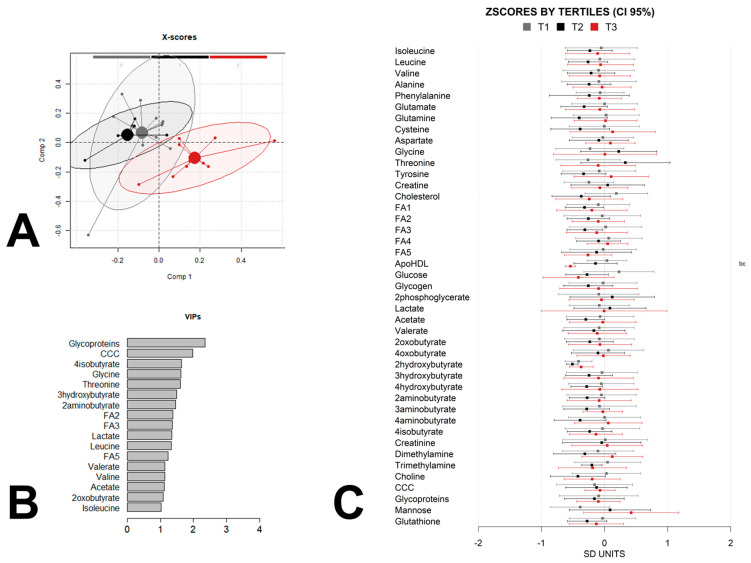
PLSDA analysis and z-scores of child metabolome at 12 months of age, by maternal-weight-gain tertile. (**A**) Score plot with 95% confidence ellipse of the PLS-DA analysis of the metabolome, adjusted for child sex, child weight at birth, maternal obesity, maternal diabetes, and maternal adherence to the Mediterranean diet for the discrimination between tertiles (tertile 1 (T1) gray, tertile 2 (T2) black, tertile 3 (T3) red). Cross-validation parameters: RMSECV 0.304, R2CV: 0.582; ROC Curve AUC: 0.96. (**B**) Metabolites with PLS-DA VIP score higher than 1 for the same PLS-DA model of the metabolome. (**C**) The Z-scores and 95% confidence intervals of individual metabolites in offspring grouped by tertiles. Metabolites with statistically significant pairwise differences between tertiles (adjusted *p*-value < 0.05) are labeled as “b” (tertile 1 vs. tertile 3), and/or “c” (tertile 2 vs. tertile 3). Label keys are the same as those in Figure 1.

To further explore the metabolic impact of maternal weight gain on offspring metabolism and identify non-linear relationships that may not have been detected by the linear regression, we split the children into groups according to the maternal-weight-gain tertiles and used partial least-squares discriminant analysis (PLS-DA) to maximize the separation between the samples and to identify discriminant patterns. We adjusted the analysis for the same confounders as the linear-regression analysis by calculating a linear-regression model with confounders for each metabolic feature and using the residues for the PLS-DA analysis. A permutation test was performed to check for the over fitting of the PLS-DA models. The multivariate chemometric models were cross-validated with 10-fold Venetian blind cross-validation. In each run, 10% of the data were left out of the training and used to test the model. Spectral regions with high variable importance in projections (VIP) coefficients obtained during PLS-DA were more critical in providing class separation during analysis. In contrast, those with very small VIP coefficients contributed less to the classification. We applied the complete PLS-DA analysis to the data at birth (Figure 2A,B), at 6 months of age (Figure 3A,B), and at 12 months of age (Figure 4A,B).

The score plot of the PLS-DA analysis of the metabolome at birth (Figure 2A) shows some moderate discrimination and a continuous trend from tertile 1 to tertile 3 of the maternal weight gain.

These trends disappeared at 6 months (Figure 3A) and 12 months (Figure 4A) of age, suggesting the influence of other factors. However, the metabolomes at 6 months of age still showed some differences between the middle tertile and the extreme tertiles. These differences disappeared again at 12 months of age in favor of a specific metabolic profile for tertile 3, indicating larger maternal weight gain. The VIPscores (Figure 2B, Figure 3B and Figure 4B) also showed that the contributions to the models of the different metabolites were different at birth and at 6 and 12 months of age. Despite these variations, some metabolites were present in all the sets of VIP scores higher than 1. Glycoprotein fragments, fatty acids, and choline-containing compounds were among the most significant contributors in all the models. Additionally, branched-chain amino acids such as isoleucine and leucine, butyrate derivatives and lactate were also present in most of the sets.

In addition to the VIP contributions, we calculated the z-scores 95% confidence intervals for each group and built logistic-regression models for pairwise group comparisons between the tertile groups which were also adjusted by the same confounders as the linear-regression models for a simpler visualization of the differences between the groups (Figure 2C, Figure 3C and Figure 4C). The analysis by tertile revealed that many of the strongest contributors to the PLS-DA models at the three time-points did not exhibit statistically significant pairwise associations between any of the three tertile groups. According to the mean differences analysis, the influence of maternal weight gain on the offspring metabolome was greatest at 6 months of age, with sixteen significant differences between tertile 1 and 2 and two differences between tertile 1 and 3, compared with the differences at birth, at which point a total of five statistically significant differences occurred, and at month 12 when only two statistically significant differences occurred, although the sample size was rather low at this time point, which may have precluded the achievement of statistical significance. At all the ages, most of the statistically significant associations corresponded to comparisons between tertile 1 and tertile 2. Interestingly, a few of these statistically significant associations expanded from birth to 6 months of age and included fatty acids, branched-chain amino acids and choline-containing compounds.

**Figure 5:**
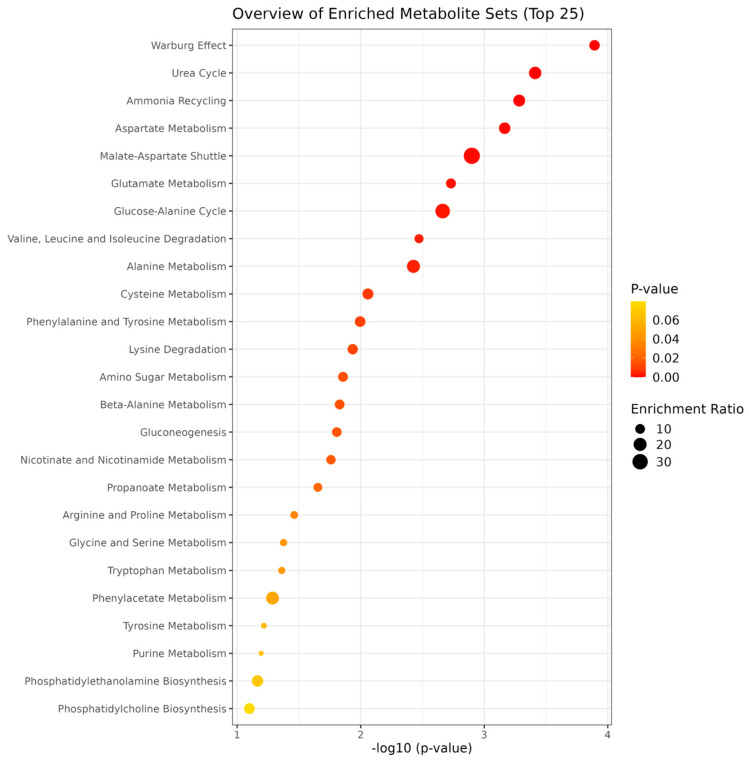
Metabolite set enrichment analysis of the association between maternal weight gain and offspring metabolome. The metabolite set enrichment analysis of metabolites with PLS-DA scores higher than 1 and adjusted *p* values < 0.05 at birth, 6 months of age, or 12 months of age. Metabolic pathways whose names are indicated are significant (*p*-values lower than 0.05 after the adjustment using the Holm-Bonferroni method and False Discovery Rate) and have a pathway impact value, calculated through pathway-topology analysis, over 0. The pathways are represented as circles. The circle colour indicates the significance level, from highest (red) to lowest (white) in the enrichment analysis. The circle size is proportional to the impact value of each pathway from the topology analysis.

Our metabolite set enrichment analysis (Figure 5) suggests that studying hypoxic-like metabolism, ketone bodies production, and ammonia-related metabolic pathways may help to understand this connection.

## 4. Discussion

Maternal GWG during pregnancy is an important determinant of fetal growth and development and can have a significant impact on the metabolic health of offspring later in life. Our study aimed to explore the metabolomic profiles of children at birth, at 6 months, and at 12 months of age, and to analyze them with respect to the maternal GWG. Although previous studies explored the metabolomic profiles of offspring and their association with maternal metabolic states, this is the first study in which the profiles are analyzed with respect to maternal GWG using both regression models and multivariate PLS-DA models adjusted for confounders. Adjusting for confounders in both the mothers and the children allowed for a more accurate detection of associations and reduced false positive discoveries. Although many metabolic associations were detected using our combined analysis, most of them were present either in the tertiles analysis or in the linear-regression analysis, suggesting a complex, nonlinear association that was probably modulated by many external factors. In fact, at birth only, the three tertiles followed a progressive trend from tertile 1 to tertile 2 and, later, tertile 3 whereas at 6 and 12 months of age, there seems to have been discrimination between the high and the low tertiles with respect to the others. The interpretation of these results is far from simple, but the score plots suggest that some metabolic shifts took place during the first 12 months of life, which may have been modulated by the maternal GWG.

The infant metabolome is influenced by a range of factors, including genetics, diet, and environmental exposures, and undergoes significant changes during the first year of life as the infant grows and develops [19]. Maternal GWG has been associated with alterations in the infant amino acid metabolome [20] and lipid metabolome [21]. The metabolites examined in this study cover a wide and rather comprehensive spectrum of compounds. By studying 43 metabolites, including amino acids, sugars, ketone bodies, lipid moieties, short-chain fatty acids, and general cellular and circulating compounds, at birth and at two more early ages, we aimed to provide a broader view of the impact of maternal weight gain during pregnancy on the infant metabolome. Our study revealed that the influence of maternal weight gain during pregnancy on the early metabolic profiles of offspring is not linear and is likely to be multifactorial. Although some differences between the tertiles of maternal weight gain appeared during birth, their influence peaked at 6 months of age, and most were resolved at 12 months of age. The impact in the long term and on other biological processes of these changes is unknown and deserves further investigation.

Among the metabolites affected by maternal weight gain in early life, butyrate derivatives and, specifically, 3-hydroxybutyrate, appear to be predominant. During the first few days of life, new-borns experience a physiological state known as neonatal ketosis, characterized by high levels of circulating ketone bodies, including 3-hydroxybutyrate. This is a normal response to the sudden cessation of glucose supply from the mother, which after a few days stabilizes to low levels. However, infants who are born prematurely may have abnormal levels of 3-hydroxybutyrate during their first year of life [19]. The organic compound 3-hydroxybutyrate has been shown to inhibit the activity of histone deacetylases, which are enzymes that remove acetyl groups from histone proteins and therefore regulate epigenetic modifications [22]. It also has important cell-signaling and regulatory functions, including pathways involved in reducing oxidative stress and inflammation, and it is potentially linked to the differences between the glycoprotein levels observed in our study [23]. Glycoproteins, which appear to be affected by maternal weight gain at birth and at 6 and 12 months, reflect leukocyte activation and are associated with inflammation. A previous study found that higher maternal weight gain during pregnancy was associated with an increase in leucocyte activation in the umbilical cord blood of newborns [24], which may be related to our findings on glycoproteins and 3-hydroxybutyrate. More recently, Jacopo et al. found lower levels of beta-hydroxybutyrate in both SGA and low-birth-weight placenta metabolomes, which may reflect poorer tolerance to hypoxia [25,26]. Finally, butyrate derivatives, such as aminobutyrates and oxobutyrates, are bacterial co-metabolites that are initially produced by the gut microbiota and further processed by human cells [27]. These facts combined with our results suggest a relevant role of this metabolite in the potential impact of maternal weight gain on infant health.

Our analysis revealed the association between other, less frequently studied, metabolites in serum and maternal weight gain. Lactate and acetate at birth appear as to be linearly correlated with maternal weight gain. In a previous study, higher maternal weight gain was associated with higher lactate levels in the cord blood of new-borns [28]. Phenylalanine, which showed statistically significant differences between tertiles at birth in our study, is an essential amino acid that is necessary for proper growth and development in infants. There is some evidence to suggest that maternal phenylalanine intake during pregnancy may be associated with increased birth weight and adiposity in offspring [29], but the underlying causes are unclear. Leucine is a branched-chain amino acid (BCAA) that is typically associated with metabolic diseases e.g., type 2 diabetes or obesity in adults [30]. However, its role in early life is controversial. In our study, the babies from mothers with lower maternal weight gain exhibit lower levels of leucine. Despite high levels of BCAAs in breast milk, the concentration of leucine and isoleucine in the blood of newborns is relatively low. Leucine levels increase rapidly during the first few months of life, reaching adult levels by around six months of age but their association with maternal weight gain and with infant health is poorly understood.

The reported levels of choline in newborns are relatively high compared to those in adults because choline is essential for brain development, which is rapid during the first few months of life [31]. By the time a baby is one year old its choline requirements are similar to those of an adult. Our results show that these findings may extend to other choline-containing related compounds, such as phosphocholine and phosphatidylcholine. Phosphocholine is also a metabolite that partially arises from the processing in the gut of dietary carnitine into trimethylamines and choline-containing compounds [32]. Our results suggest that maternal weight gain during pregnancy affects the bacterial ecosystem of the baby. Microbial co-metabolism can affect nutrient availability and absorption, as well as immune-system development, and may be pivotal in many of the metabolic effects we observed in this study. While the initial colonization of the gut microbiota is strongly influenced by the mother’s microbiota during delivery and breastfeeding, the microbiota evolves and becomes more diverse based on a variety of factors throughout life.

On one hand, the main strength of our study lies in the novelty of its analysis of offspring metabolomic profiles according to maternal GWG and their adjustment according to other common confounders of both the mother (obesity, diabetes during gestation and adherence to the Mediterranean diet) and the child (sex, weight at birth and type of feeding). Moreover, this analysis was not limited to the moment of birth, but instead continued up to the age of one year, identifying what may be markers of cardiovascular risk later in life.

On the other hand, our study has several limitations. First, the high cost of metabolomic studies and the difficulty in obtaining subjects has caused the initial sample size to be modest, which could have played a role in the failure to find clinical differences and in the missing of some metabolomic differences between the three groups. Second, the progressive loss of subjects during follow-up, which was particularly affected by the COVID pandemic, lmeant that the sample sets at different ages (at 6 months and, especially at 12 months of age) had a smaller number of subjects. Finally, we also lacked information about other potential co-variables such the infant microbiome, the exact duration of exclusive breastfeeding, the total formula intake, and the order of introduction and quantity of other complementary foods.

Overall, the mechanisms underlying the relationship between maternal weight gain and metabolic profiles in infants are not well understood, but it has been hypothesized that higher maternal weight gain may lead to alterations in placental function and metabolism, which could in turn affect fetal and infant metabolism. We identified several metabolites associated with maternal weight gain either linearly or non-linearly by tertiles which could help to identify metabolic pathways and clusters involved in long-term implications. Our metabolite set enrichment analysis (Figure 5) suggests that studying hypoxic-like metabolism, ketone bodies production, and ammonia-related metabolic pathways may help to understand this connection. The presence of high amino derivatives of short-chain fatty acids, methylamines, and choline-containing compounds in all our significant metabolic sets suggests the relevant role of nitrogen metabolism in this network of interactions. Further research is needed to elucidate the mechanisms through which maternal GWG modulates these changes in children’s metabolome, as well as the long-term effects in later life. It is very difficult to translate these metabolomic findings into clinical routine with our current knowledge, but further investigation may help to identify at-risk infants for whom to target more personalized dietary and growth monitoring interventions.

## Figures and Tables

**Table 1:** Characteristics of mothers, according to absolute maternal GWG tertiles. Data are presented as either mean ± SD or no. (%). ǂ Analysis of variance (ANOVA). ¥ The Kruskal Wallis test. • Chi-squared test. Data with significant *p*-values are labeled as follows: ** for *p* < 0.01 and *** for *p* < 0.001.

Mothers’ Variables	1st Tertile GWG (N = 28)	2nd Tertile GWG (N = 27)	3rd Tertile GWG (N = 28)	*p* Value
Age (years) ¥	31.5 (± 8.48)	34.11 (± 5.17)	33.18 (± 5.41)	0.429
Maternal obesity	Yes 75% No 25%	Yes 48.1% No 51.9%	Yes 57.1% No 42.9%	0.116
BMI prior to pregnancy (Kg/m²) ¥	33.61 (±7.31)	28.34 (± 7.07)	28.07 (± 6.03)	0.004 **
GWG (Kg) ǂ	2.33 (± 4.31)	10.11 (± 1.12)	16.78 (± 3.91)	<0.001 ***
GWG adequacy according to IOM recommendations •	Yes 100% No 0%	Yes 66.7% No 33.3%	Yes 21.4% No 78.6%	<0.001 ***
AMD score ¥	6.96 (± 2)	7.76 (± 1.7)	7.46 (± 1.6)	0.396
Degree of AMD •	High 12.5% Medium 58.3% Low 29.2%	High 23.5% Medium 70.6% Low 5.9%	High 8.3% Medium 75% Low 16.7%	0.266
Diabetes during pregnancy	Yes 35.7% No 64.3%	Yes 11.1% No 88.9%	Yes 17.9% No 82.1%	0.072
Smoking habits •	Yes 14.3% No 85.7%	Yes 18.5% No 81.5%	Yes 21.4% No 78.6%	0.78
Type of delivery •	Vaginal 50% Cesarean 50%	Vaginal 44.4% Cesarean 55.6%	Vaginal 60.7% Cesarean 39.3%	0.469

**Table 2:** Characteristics of children at birth, according to absolute maternal GWG tertiles. Data are presented as either mean ± SD or no. (%). ǂ Analysis of variance (ANOVA). ¥ The Kruskal Wallis test. • Chi-squared test. Data with significant *p*-values are labelled as follows.

Newborns’ Variables	1st Tertile GWG (N = 28)	2nd Tertile GWG (N = 27)	3rd Tertile GWG (N = 28)	*p* Value
Birth weight (BW) (g) ǂ	3363 (±552)	3311 (±557)	3454 (±449)	0.593
Weight percentile ǂ	53.45 (±31.14)	52.4 (±31.1)	58 (±30.4)	0.773
BW Classification •	SGA 7.1% AGA 75% LGA 17.9%	SGA 11.1% AGA 70.4% LGA 18.5%	SGA 10.7% AGA 60.7% LGA 28.6%	0.8
Length (cm) ǂ	49.37 (±2.08)	48.6(±1.94)	49.67 (±2.13)	0.149
Length percentile ǂ	48.81 (±28.2)	39.78 (±25.71)	53.33 (±31.8)	0.21
Head circumference (HC) (cm) ¥	34.46 (±1.52)	34.38 (±1.57)	34.97 (±1.41)	0.351
HC percentile¥	62.8 (±27.01)	64 (±28.79)	71.68 (±27.67)	0.323
ponderal index ¥	2.77 (±0.267)	2.85 (±0.24)	2.81 (±0.31)	0.406
Systolic blood pressure (mmHg) ǂ	78.63 (±87)	81.5 (±12.5)	78.89 (±12.66)	0.605
Diastolic blood pressure (mmHg) ǂ	50.66 (±10)	49.79 (±9.63)	49.5 (±9.45)	0.899
Heart rate (bpm) ǂ	126.81 (±14.69)	129.65 (±12.65)	132.69 (±16.93)	0.366
Type of feeding •	breast 67.9% formula 32.1%	breast 88.9% formula 11.1%	breast 75% formula 25%	0.169
Formula Supplementation •	Yes 31.6% No 68.4%	Yes 29.2% No 70.8%	Yes 19% No 81%	0.62

**Table 3:** Characteristics of children at 12 months of life according to tertiles of absolute maternal GWG. Data are presented as either mean ± SD. ǂ Analysis of variance (ANOVA). ¥ The Kruskal Wallis test. Data with significant *p*-values are labeled as follows: * for *p* < 0.05.

	1st Tertile GWG	2nd Tertile GWG	3rd Tertile GWG	*p* Value
**12 Month Somatometry**	(N = 27)	(N = 24)	(N = 25)	
Weight (g) ǂ	10,509 (±1516)	9736 (±1193)	10,293 (±1242)	0.114
Weight percentile ¥	71.14 (±28.75)	58.54 (± 29.43)	61.92 (± 27.87)	0.214
Length (cm) ǂ	76.74 (±3.4)	74.83 (±2.64)	76.64 (±2.4)	0.039 *
Length percentile ¥	60.4 (±32)	48.79 (±28.72)	60.08 (±26.68)	0.128
Head circumference (HC) (cm) ¥	46.38 (±1.32)	45.81 (±1.15)	46.80 (±1.69)	0.094
HC percentile ¥	64.64 (±27.89)	57.83 (±24.94)	68.2 (±29.1)	0.216
BMI (Kg/m²) ǂ	17.65 (±1.53)	17.29 (±1.76)	17.38 (±1.69)	0.722
BMI percentile ¥	67.86 (±29.172.63)	6(±28.24)	60.07(±29.59)	0.516
**12 Month** **Blood Test**	(N = 12)	(N = 8)	(N = 12)	
Leptin (pg/ml) ¥	452 (±613)	691 (±380)	513 (±626)	0.378
Total cholesterol (mg/dl) ǂ	160.6 (±32)	158 (±38.2)	152.75 (±25.64)	0.827
HDL cholesterol (mg/dl) ǂ	40.5 (±9.4)	44.75 (±10.91)	43.16 (±10.66)	0.64
LDL cholesterol (mg/dl) ǂ	95.75(±34.6)	100.2 (±33.8)	96.5 (±20.5)	0.94
Triglycerides (mg/dl) ǂ	183 (±90)	173 (±90.7)	119 (±45)	0.115
HOMA index ¥	0.955 (±0.77)	1.99 (±1.58)	1.34 (±1.15)	0.126
Uric acid (mg/dl) ǂ	3.15 (±0.64)	3.22 (±0.49)	3.05 (±0.669)	0.822

## Data Availability

The data presented in this study are available on request from the corresponding author. The data are not publicly available due to ethical reasons.
